# Prognostic and Predictive Models in Myelofibrosis

**DOI:** 10.1007/s11899-024-00739-6

**Published:** 2024-08-24

**Authors:** Barbara Mora, Cristina Bucelli, Daniele Cattaneo, Valentina Bellani, Francesco Versino, Kordelia Barbullushi, Nicola Fracchiolla, Alessandra Iurlo, Francesco Passamonti

**Affiliations:** 1 Fondazione IRCCS Ca’ Granda Ospedale Maggiore Policlinico, Via Francesco Sforza, 35 – 20122, Milan, Italy; 2 Dipartimento Di Oncologia Ed Onco-Ematologia, Università Degli Studi Di Milano, Via Francesco Sforza, 35 - 20122, Milan, Italy

**Keywords:** Myelofibrosis, Prognosis, Next generation sequencing, Allogenic-stem cells transplant, JAK inhibitors

## Abstract

**Purpose of Review:**

Myelofibrosis (MF) includes prefibrotic primary MF (pre-PMF), overt-PMF and secondary MF (SMF). Median overall survival (OS) of pre-PMF, overt-PMF and SMF patients is around 14 years, seven and nine years, respectively. Main causes of mortality are non-clonal progression and transformation into blast phase.

**Recent Findings:**

Discoveries on the impact of the biological architecture on OS have led to the design of integrated scores to predict survival in PMF. For SMF, OS estimates should be calculated by the specific MYSEC-PM (MYelofibrosis SECondary-prognostic model). Information on the prognostic role of the molecular landscape in SMF is accumulating. Crucial treatment decisions for MF patients could be now supported by multivariable predictive algorithms. OS should become a relevant endpoint of clinical trials.

**Summary:**

Prognostic models guide prediction of OS and treatment planning in MF, therefore, their timely application is critical in the personalized approach of MF patients.

## Introduction

Myelofibrosis (MF) is a BCR::ABL1-negative myeloproliferative neoplasm (MPN) characterized by splenomegaly, constitutional symptoms, heterogeneous blood cell alterations and bone marrow fibrosis (BMF). MF also presents an inherent tendency to evolve into blast phase (BP) [1, 2]. MF encompasses primary MF (PMF), which includes prefibrotic- (pre) and overt-PMF, and secondary MF (SMF), in case of a previous diagnosis of polycythemia vera (PV) or essential thrombocythemia (ET) [1–5].

MF is a rare neoplasm, with an incidence of 0.44/100000 patients per year in US by a recent report [6]. Median age at MF onset is in the seventh decade [6], but it could also affect younger patients, who need an accurate prognostic assessment for possible selection to allogeneic hematopoietic stem cells transplant (allo-SCT).

MF is characterized by phenotypic driver gene mutations involved in the downstream activation of the JAK-STAT pathway [1, 7]. Two-thirds of patients with PMF harbor *JAK2*V617F, 25% *CALR*, and 10% each *MPL* or no driver mutation (‘triple negative’ status, TN) [8]. Almost all post PV (PPV-) MF carry *JAK2* mutations, while near half post ET (PET-) MF patients show *JAK2*V617F, 30% *CALR* and 5–10% *MPL* mutations or TN status [9]. *CALR* mutations are distinguished in type 1/type 1(-like) and type 2/type 2(-like), respectively a deletion of 52 base pairs (bp) and an insertion of five bp (or similar alterations). Of note, *CALR* and *MPL* mutations could be found in 30% of ET and MF cases with low (< 5%) *JAK2*V617F variant allele frequency (VAF), with “double mutated” subjects showing higher platelets *vs* those with just low *JAK2*V617F VAF [10].

Additional non-driver myeloid neoplasms-associated gene variants (M-GVs) have been identified in MF [8]. About 80% of PMF and 69% of SMF cases carry M-GVs, respectively [8, 11, 12]. Involved genes are related to epigenetic modifiers (*DNMT3A, TET2* and *ASXL1*), splicing factors (*SF3B1, SRSF2* and *U2AF1*), metabolic enzymes (*IDH1* and *IDH2*), and tumor suppressors (*TP53*) [8, 11, 12]. Information on the prevalence and impact of M-GVs in MF is accumulating, due to the increasing diffusion of methods such as *Next generation sequencing* (NGS).

Patients affected by MF have a significantly reduced outcome, with a median OS of 14 years, seven and nine years in pre-PMF, overt-PMF and SMF, respectively [13–15]. Both in PMF and in SMF cohorts, non-clonal progression accounts for around one third of deaths [13, 14], including second malignancies [16], infections and cardiovascular events [2]. Evolution into BP occurs in 10–15% of MF cases, with a severely reduced OS [14, 17].

Nonetheless, in recent years improvement in prognosis has been registered. A retrospective study compared 844 MF patients by decade of presentation: 2000–2010 and 2011–2020 [18]. In the latter decade, reported median OS was significantly higher (63 *vs* 48 months), even in cases with unfavourable features [18]. This is due to a greater awareness of disease, a better insight on the biological background, the widespread use of JAK inhibitors (JAKis), improved supportive care, and a more accurate selection and management of candidates to allo-SCT [18–23].

On the contrary, the outcome of post-MF BP still remains dismal, representing an unmet clinical need. Risk factors for BP have been extensively investigated and are outside the scope of this review [24, 25]. Of note, our group recently described a wide cohort of PMF and SMF patients, confirming the predictive role of anemia, also while on JAKis treatment [25].

Conventional evaluation of prognosis in MF is based on symptoms, demographic and hematologic data [13, 26]. However, the increasing knowledge on the biological landscape has led to the design of integrated prognostic models, now of widespread use mainly in PMF. Unfortunately, real world (RW) data show that around 40% of MF patients still receive an inaccurate risk definition and one third of cases any categorization at all [27].

In this review, we will focus on the evolving paradigm of survival definition in PMF and SMF over the years. We will also underline the importance of a correct selection of potential candidates to allo-SCT. Then, recent insights on the outcome with JAKis and innovative drugs will be presented. All this information will guide treating physicians to a personalized approach of individuals affected by MF.

## Primary Myelofibrosis: How to Make the Best Use of Multiple Prognostic Scores

The 2009 *International Prognostic Scoring System* (IPSS) represents still nowadays the most used prognostic score at time of PMF diagnosis [26]. Variables included are age > 65 years, hemoglobin (Hb) < 10 g/dL, leukocyte count > 25 × 10^9/L, circulating blasts ≥ 1%, and constitutional symptoms [26]. Every parameter has been scored one point [26]. Median OS of the four IPSS categories (low, intermediate-1, intermediate-2 and high risk) spans between 11.3 and 2.3 years [26].

In 2011, the *Dynamic IPSS* (DIPSS) has been developed to be applied at any time during follow-up [13]. DIPSS includes the same variables of IPSS, but the prognostic weight of anemia is higher (Table 1) [13]. Subjects are divided into four risk groups, with intermediate-2 and high risk categories having a median OS of less than five years (Table 1) [13].

**Table 1:** Prognostic models for patients with primary and secondary myelofibrosis

	DIPSS	DIPSS^+^	MIPSS-70	MIPSS-70^+^ version 2.0	MYSEC-PM
*Patients’ characteristics (score)*	Age > 65 y (1) Constitutional symptoms (1)	Age > 65 y (1) Constitutional symptoms (1) RBC transfusions need (1)	Constitutional symptoms (1)	Constitutional symptoms (2)	Age (0.15 × y) Constitutional symptoms (1)
*Laboratory values (score)*	Hb < 10 g/dl (2) WBC > 25 × 10^9/l (1) Blasts ≥ 1% (1)	Hb < 10 g/dl (1) WBC > 25 × 10^9/l (1) Blasts ≥ 1% (1) PLT < 100 × 10^9/l (1)	Hb < 10 g/dl (1) WBC > 25 × 10^9/l (2) Blasts ≥ 2% (1) PLT < 100 × 10^9/l (2)	Severe anemia^2^ (2) Moderate anemia^3^ (1) Blasts ≥ 2% (1)	Hb < 11 g/dl (2) Blasts ≥ 3% (2) PLT < 150 × 10^9/l (1)
*Driver mutation* *(score)*			Absence of type 1/like *CALR* (1)	Absence of type 1/like *CALR* (2)	Absence of *CALR* (2)
*Myeloid-gene variants* *(score)*			1 HMR (1) ≥ 2 HMR (2)	1 HMR included *U2AF1*Q157 (2) ≥ 2 HMR included *U2AF1*Q157 (3)	
*Karyotype* *(score)*		Unfavourable^1^ (1)		Unfavourable^4^ (3) Very high-risk^5^ (4)	
*Bone marrow (score)*			BMF grade ≥ 2 (1)		
***Risk (score*),** median survival	*Low (0),* NR *Int-1 (1–2),* 14.2 y *Int-2 (3–4),* 4 y *High (5–6),* 1.5 y	*Low (0),* 15.4 y *Int-1 (1),* 6.5 y *Int-2 (2–3),* 2.9 y *High (*≥ *4),* 1.3 y	*Low (0–1),* NR *Int (2–4),* 6.3 y *High (*≥ *5),* 3.1 y	*Very low (0),* NR *Low (1–2),* 16.4 y *Int (3–4),* 7.7 y *High (5–8),* 4.1 y *Very high (*≥ *9),* 1.8 y	*Low (*< *11),* NR *Int-1 (11–13),* 9.3 y *Int-2 (14–15),* 4.4 y *High (*≥ *16),* 2 y

DIPSS = Dynamic International Prognostic Scoring System; MIPSS = Molecular Enhanced International Prognostic Score System; MYSEC-PM = MYelofibrosis SECondary to polycythemia vera and essential thrombocythemia-Prognostic Model; y = years; RBC = red blood cells; Hb = hemoglobin; WBC = white blood cells; PLT = platelets; HMR = high molecular risk (one among *ASXL1, EZH2, SRSF2,* or *IDH1/2*); BMF = bone marrow fibrosis; NR = not reached; Int = intermediate

^1^ = complex karyotype or sole or two abnormalities including + 8, -7/7q-, i(17q), -5/5q-, 12p-, inv(3) or 11q23 rearrangement

^2^ = Hb < 8 g/dl in women, Hb < 9 g/dl in men

^3^ = Hb 8–9.9 g/dl in women, Hb 9–10.9 g/dl in men

^4^ = chromosomal abnormalities except “very high-risk” (see below) or sole 13q-, + 9, 20q-, chromosome 1 translocation/ duplication or sex chromosome alterations including -Y

^5^ = single/multiple abnormalities of -7, i(17q), inv(3)/3q21,12p-/12p11.2,11q-/11q23, + 21, or other autosomal trisomies except + 8/9

The DIPSS^+^ model is a revised version of the DIPSS [28], that considers also red blood cell (RBC) units need, platelet (PLT) count, and karyotype (Table 1) [28, 29]. Of note, only patients with overt-PMF were considered when the overmentioned scores were developed, and it has been shown that IPSS could not discriminate pre-PMF patients well [15, 30].

Among driver mutations, *CALR* type 1 has been associated with the most favorable outcome in PMF [15, 31]. As for M-GVs, abnormalities in *ASXL1* are the most frequent (30% of patients) [32]. Together with *ASXL1,* M-GVs in *SRSF2*, *EZH2*, and *IDH1*/*IDH2* were defined as high molecular risk (HMR) mutations, since they were correlated with reduced OS and increased risk of BP [32]. The impact of HMR alterations depends also on their number [15, 32, 33]. *CALR* type 1(-like) and HMR mutations have been integrated in the *Mutation-enhanced IPSS-70* (MIPSS-70) model, developed for potential candidates to allo-SCT (subjects ≤ 70 years) (Table 1) [33]. In this cohort, 80% of patients showed *CALR* type 1 wild type, 31–41% and 8–9% at least one or more HMR alterations, respectively [33]. The MIPSS-70 includes also histological features, underlying the relevance of at least grade 2 BMF (overt-PMF) compared to less than grade 2 (pre-PMF) [33]. Median OS of the high risk MIPSS-70 group is below five years (Table 1) [33].

The MIPSS-70 was lately revised in the MIPSS-70^+^ model, that included information on unfavorable cytogenetics, the latter defined as any abnormal karyotype (AK), except for sole abnormalities of 20q-, 13q-, + 9, chromosome 1 translocation/duplication, -Y, or sex chromosome abnormality excluding -Y [33]. Of note, both MIPSS-70 and MIPSS-70^+^ scores appear also able to discriminate the mortality risk of patients above 70 years [33].

A further revision, the MIPSS-70^+^ version 2.0, encompasses *U2AF1*Q157 among HMR mutations, includes sex- and severity-adjusted anemia cut-offs and a so-called “very high” cytogenetic risk group (the latter detailed in Table 1) [34]. On the other side, information on BMF grade, leukocyte and PLT count has been omitted. Patients in the high and very-high risk MIPSS-70^+^ version 2.0 groups have an estimated OS below five years [34]. The *Genetically Inspired Prognostic Scoring System* (GIPSS) considers only cytogenetic and molecular information, as the absence of *CALR* type 1(-like) and the presence of *ASXL1*, *SRSF2* or *U2AF1*Q157 mutations [35]. Predictive power of this model was suggested by the Authors to be comparable to that of MIPSS-70^+^ [35].

The updated *National Comprehensive Cancer Network* (NCCN) guidelines (version 1.2024) suggest applying in PMF the prognostic models reported in Table 1 [36], based on the type of available information: the MIPSS-70 or MIPSS-70^+^ version 2.0 for molecularly annotated cases, the DIPSS^+^ if absent molecular data but known karyotype, or the DIPSS if even cytogenetics is unavailable.

In the clinical practice, we calculate all the overmentioned scores (Table 1) at the same time [2]: DIPSS is easy and quick to calculate, but time-requiring data on BMF grade and M-GVs are particularly relevant. In our opinion, the high frequency of “dry tap” in PMF is the main limit to cytogenetics-based models, but this data might be obtained on peripheral blood.

To simultaneously calculate these models, a PMF-specific web calculator has been recently proposed by our group: https://pmfscorescalculator.com [2]. We are aware that this practice might lead to discordant mortality estimates among scores [2]: in such cases, we suggest a close follow-up of the patient, for detecting early signs of disease progression and possible indication to allo-SCT [2].

Besides the over mentioned well-structured models, several other factors have been correlated with outcome in PMF.

Among hematological variables, a “myelodepletive” phenotype (the presence of at least one cytopenia) was associated with a shorter OS in univariate analysis [37]. To overcome the poorly standardizable definition of circulating blasts by morphology, the application of flow cytometry seems to improve the accuracy of the MIPSS-70 [38].

An attempt was made to fit comorbidities into conventional models, but results are not definitive to date [39, 40].

Information on the prognostic impact of M-GVs is accumulating. The French group proposed a “NGS model” that distinguishes four genetic groups [41]: *TP53*, “High risk” (≥ 1 mutation in *EZH2, CBL, U2AF1, SRSF2, IDH1* and *IDH2*), *ASXL1-*only and “Others” mutations. In this analysis, *ASXL1* alterations were associated with an unfavorable outcome only if they co-occurred with *TP53* or “High risk” M-GVs [41]. On the contrary, applying the same NGS categories to another independent cohort of PMF cases [42], those mutated for *TP53* or for “High risk” genes displayed the worst OS, but also *ASXL1* mutated-only group had a clearly reduced outcome with respect to the “Others” [42]. *ASXL1* mutations co-occurred in two thirds of “High risk” cases and implied a worse OS [42]. A recent study by the Spanish group pointed out the independent relevance of *ASXL1* VAF > 20%, more than gene mutation per se [43]*.* Alterations in RAS/MAPK pathway genes have been related with an unfavorable OS in overt-PMF [44, 45]. However, the integrity of the MIPSS-70 variants was not significantly upgraded by the inclusion of RAS/MAPK mutations, as of *TP53* and *RUNX1* alterations [45]. Besides, the low incidence of those M-GVs will require an external validation for confirming their negative impact [45]. Of note, *ASXL*, *IDH1/2*, *N/KRAS*, *U2AF1* and *CUX1* alterations are enriched within the overmentioned “myelodepletive” phenotype [37]. Very recently, a Spanish collaborative study has proposed to apply artificial intelligence (AI) methods for integrating NGS and clinical data to better define outcome [46].

From a biological point of view, levels of the neutrophil chemoattractant CXCL8 are increased in PMF and negatively correlate with OS [47]. An Italian collaborative group evaluated the expression of 201 genes in granulocytes of MF patients, identifying outcome-related transcripts [48]. Subjects with pre-PMF were characterized by a “low risk” gene espression (GE) signature, with more favourable OS and BP-free survival [48]. The same group demonstrated the increased expression profile of a set of circulating long non-coding RNAs (lncRNAs), that appeared to be evaluable biomarkers of unfavorable outcome [49]. In CD34 + hematopoietic stem/progenitor cells from MF, reactive oxygen species (ROS) levels correlate with shorter OS [50].

## Secondary Myelofibrosis: How to Specifically Define Prognosis

In a recent meta-analysis of over 3.000 PV patients treated with hydroxyurea (HU), the rate of SMF was 0.9%, 5% and 33.7% at 1, 5 and 10 years, respectively [51]. Out of 576 ET subjects, 9.5% evolved into SMF after 15 years of follow up [17].

Median time to transformation into SMF seems to be related to the type of driver mutation [52]: in a multivariable model, patients with *CALR* mutated ET had a significantly longer time to progression compared to *JAK2* mutated ET/PV and, even more, to TN cases [52].

Predictive factors for evolution of PV or ET cases to SMF have been extensively investigated [24]: clinical features, cytogenetic alterations, bone marrow (BM) characteristics, driver mutations type and VAF, M-GVs and dysregulation of biological pathways are involved. All this information could therefore lead to a personalized monitoring of PV and ET patients [24].

Among cytoreductive therapies, only some retrospective data suggested a protective role of interferons [53, 54]. More relevant appears the impact of long-term treatment with ruxolitinib (RUX) in PV cases resistant or intolerant to HU [55, 56]. Both in the prospective randomized phase 2 MAJIC-PV trial and in an Italian RW experience, the achievement of at least a partial molecular response (≥ 50% reduction in *JAK2*V617F VAF) was correlated to a significantly longer SMF-free survival [55, 56].

When a diagnosis of SMF is established [1], OS estimate could not be properly calculated by the prognostic models used for PMF [57, 58]. In 2014, an international study focused just on SMF cases, called the *MYelofibrosis SECondary to PV and ET* (MYSEC) project, was started [9].

The original database retrospectively included 685 PPV- and PET-MF cases annotated for driver mutations [9]. Median OS was 9.3 years for the whole dataset, with a borderline difference between the two subtypes (14.5 *vs* 8.1 years for PET- and PPV-MF) [9]. In a multivariable analysis, *CALR* mutated patients had a better outcome compared to *JAK2*V617F mutated SMF [9].

The MYSEC cohort, that represents to date the largest dataset of SMF patients, allowed to generate a specific clinical-molecular prognostic score, the *MYSEC-Prognostic Model* (MYSEC-PM) [14]. As reported in Table 1, the higher prognostic weight was assigned to anemia, increased blasts count and absence of *CALR* alterations [14]. Four MYSEC-PM risk categories were identified, with intermediate-2 and high risk cases having a median OS below five years [14]. The MYSEC-PM could be easily calculated by a nomogram depicted on the original paper and by an online application (https://mysec.shinyapps.io/prognostic_model/) [14]. Even though this score was established at time of SMF diagnosis, it has also been dynamically validated [59].

Other prognostic factors have been investigated in SMF.

Similar to PMF models, also the MYSEC-PM includes blast count by morphology as a variable [14]. Differently from PMF, integrating flow cytometry results did not outperform the standard MYSEC-PM counterpart [38]. In the MYSEC database, female patients showed a better OS, also considering age at SMF diagnosis [60]. This is in line with data on large cohorts of ET subjects [61], while the prognostic relevance of gender in PV is still a matter of debate [62].

The impact of BMF (grade 2 *vs* 3) has been investigated in a more recent subanalysis of the MYSEC cohort [63]: out of 805 SMF, 34% had a grade 3 BMF at evolution. In univariate analysis, this latter cohort had a significantly lower OS compared to patients with grade 2 BMF (7.4 *vs* 8.2 years), underlying the importance of performing a BM biopsy to confirm SMF [63].

Around one third of 376 cytogenetic-annotated MYSEC cases had an AK [64]. Those subjects had a significantly reduced outcome compared to normal karyotype (NK): the median OS was 6.1 *vs* 10.1 years [64]. Of note, patients with monosomal karyotype (MK), complex karyotype (CK) without MK and those with CK had an estimated OS below 3.5 years [64]. Integrating cytogenetics did not improve the prognostic power of the MYSEC-PM, nonetheless we suggest assessing it in case of suspected SMF evolution [64]. Recently, *Shide *et al*.* have applied the DIPSS^+^ model to a cohort of Japanese SMF patients, and they showed a better outcome prediction compared to the MYSEC-PM [65]. Of note, their study included just 183 cases [65].

As for M-GVs, information in SMF is accumulating. Looking at HMR, *Rotunno *et al*.* confirmed the unfavorable prognostic role only of *SRSF2* in PET-MF [66]. Applying the over mentioned “NGS model” to 193 SMF cases, *TP53* mutations conferred the worst outcome (median OS, 13 months), while the prognosis of *ASXL1* mutated-only patients was similar to the “Others” and the “High-risk” groups (median OS of 141, 131 and 58 months, respectively) [41, 42]. *ASXL1* mutations were detected in over half of “High-risk” subjects, without influencing their outcome [42]. Another group suggested that the performance of the MIPSS-70^+^ version 2.0 might be superior to the MYSEC-PM (C-index 0.79 *vs* 0.73), but only 155 SMF patients were included [67]. Besides, looking at OS estimates in that analysis, MIPSS-70^+^ version 2.0 recognized only three out of 58 patients with median OS below five years (so candidates for allo-SCT indication), finally limiting the usefulness of the model in the setting of SMF [67]. *Loscocco *et al*.* showed that alterations of the splicing factor *SF3B1*, found in 5% of 195 SMF patients, could be related to reduced OS [68].

*Mora *et al*.* reported the preliminary results of 639 NGS-annotated MYSEC cases [12]: around 69% of the cohort presented at least one M-GV. Among the latter, 31% and 18% showed two and at least three alterations [12]. The most frequent (≥ 10%) M-GVs interested *ASXL1, TET2, DNMT3A* and *TP53* [12]. The number of M-GVs appeared to be prognostically relevant in univariate analysis: subjects without them had a median OS significantly longer than cases with any alteration (14.8 *vs* 11.8 years) [12]. Of note, patients with at least three M-GVs had a remarkably reduced outcome compared to those with at most two mutations (median OS, 8.6 *vs* 14.8 years) [12]. In our opinion, AI methods should be applied to identify the most significant variables for integrated models [69, 70].

From a biological point of view, the overmentioned “high risk” GE signatures were enriched in PPV/PET-MF cases [48]. Similar to PMF, a set of lncRNAs with unfavorable impact on outcome was more frequently expressed [49]. Besides, high plasma levels of ROS were found to be a surrogate of shorter OS [50].

## Allogenic Hematopoietic Stem Cells Transplant: How to Select the Best Candidates

To date, allo-SCT is the only curative treatment for MF patients [71]. When evaluating possible candidates, patients’ age is not considered a limit per se [72]. More important is an accurate estimation of the outcome related not only to MF biology, but also to possible allo-SCT complications [73].

Very recently, updated recommendations by the European Society for Blood and Marrow Transplantation/European LeukemiaNet (EBMT/ELN) International Working Group were published, in light of contemporary management of MF patients [23]. It is acceptable to candidate fit subjects younger than 70 years, with an expected OS below five years, i.e., intermediate-2 and high risk DIPSS/MYSEC-PM or high risk MIPSS-70(^+^) [23]. Cases with intermediate-1 DIPSS or intermediate MIPPS-70(^+^) should be discussed, balancing patients’ preferences, available treatment alternatives, and other risk features, i.e., multi-hit *TP53* mutations, that have been associated with increased risk of BP [23, 74]. DIPSS was also judged useful for defining the timing of allo-SCT [23].

Once a potential candidate has been selected through these criteria, two other scores should be applied at referral to allo-SCT, with the aim of predicting subsequent outcome [75, 76]. *Gagelmann *et al*.* described the *Myelofibrosis Transplant Scoring System* (MTSS), that considers driver mutation, *ASXL1* variant, age, performance status, PLT and leukocyte count, and type of donor (Table 2) [75]. Of note, the MTSS was proposed and validated both in PMF and SMF, but the impact of *ASXL1* in SMF is yet to be cleared.

**Table 2:** Predictive models for allogenic hematopoietic stem cells transplant outcome in myelofibrosis

	MTSS	CIBMTR/EBMT
*Patients’ characteristics* *(score)*	Age ≥ 57 y (1) Karnofsky < 90% (1) MMUD (2)	Age > 50 y (1) MMUD (2) MUD (1)
*Laboratory data* *(score)*	WBC > 25 × 10^9/l (1) PLT < 150 × 10^9/l (1)	Hb < 10 g/dl (2)
*Driver mutation (score)*	Absence of *CALR/MPL* (2)	
*Myeloid-gene mutations (score)*	*ASXL1* (1)	
*Risk category (score*), survival^1^	*Low (0–2),* 90% *Int (3–4),* 77% *High (5),* 50% *Very high (6–9), 34%*	*Low (0–2),* 69% *Int (3–4),* 51% *High (5),* 34%
*Risk category (score*), TRM	*Low (0–2),* 10% *Int (3–4),* 22% *High (5),* 36% *Very high (6–9), 57%*	*Progressively increasing with higher scores*

MTSS = Myelofibrosis Transplant Scoring System; CIBMTR = Center for International Blood and Marrow Transplant Research; EBMT = European Society for Blood and Marrow Transplantation; y = years; Hb = hemoglobin; WBC = white blood cells; PLT = platelets; MMUD = mismatched unrelated donor; MUD = matched unrelated donor; Int = intermediate; TRM = transplant related mortality

^1^ = 5-years survival for MTSS; 3-years survival for CIBMTR/EBMT

Within the MTSS, the median 5-year OS ranged between 90% and 34%, while in the same time frame allo-SCT related mortality (TRM) varied, inversely, from 10% to 57% [75]. Based on this data, the updated EBMT/ELN guidelines suggest considering low and some intermediate risk MTSS patients for allo-SCT [23]. *Tamari *et al*.* developed an easier model in a setting without molecular testing (Table 2) [76]: age, type of donor and Hb levels at time of allo-SCT influenced both the 3-year OS probability and TRM [76].

Impact of donor type in MF is well known: out of 233 cases, the 5-year OS after allo-SCT was 56% with matched sibling, 48% with matched unrelated, and 34% with partially matched/mismatched unrelated donors [71].

We are aligned with current EBMT/ELN guidelines indications [73], but we also believe that a more personalized selection will derive applying integrated statistical methods, to identify different clinical-genomic subgroups [69, 70].

## JAK Inhibitors and Investigative Drugs: How to Read Data on Survival

Data on the impact of JAKis on outcome should be interpreted considering that OS did not represent the primary endpoint of related clinical trials, and that matched-controlled, or population-based studies have some limitations [2]. At present, most of the evidence concerns RUX [18–20, 24, 77].

Long-term pooled analysis of the COMFORT-I/II studies showed a 30%-reduction in risk of death in intermediate-2/high risk MF *vs* controls [20, 78, 79]. Moreover, 4-years OS was significantly longer (63% *vs* 57%) if RUX was started within one year from diagnosis compared to after the first 12 months, favouring an early initiation of treatment [80]. RW data with appropriate follow-up came from the ERNEST (*European Registry for Myeloproliferative Neoplasms: Toward a Better Understanding of Epidemiology, Survival, and Treatment*) project, where the outcome was significantly improved in patients treated with RUX compared to HU (median OS, 6.7 *vs* 5.1 years) [81]. This difference was even more evident in a propensity score-matching analysis, that anyway regarded only a small subgroup [81].

We previously reviewed factors impacting OS in RUX-treated patients [24]: baseline prognostic risk and blasts count, M-GVs, spleen response and RBC transfusions play a role [82–90]. More recently, *Kuykendall *et al. showed that changes in albumin levels are associated with OS [91].

Around half of patients discontinue RUX at 3 years, mostly for progression or intolerance, with a subsequent dismal outcome [92, 93]. To early identify subjects that could benefit from a prompt treatment shift, we investigated predictors of OS collected during the first six months of RUX [85]. This collaborative effort led to the design of a new prognostic model, named *Response to Ruxolitinib After 6 Months* (RR6, easily computable at http://www.rr6.eu/) [85]. This score can distinguish three categories with different OS after 6 months of RUX treatment based on the changes of RUX dose and of spleen length, and on the need of RBC units during the same period (Table 3) [85]. Based on the RR6 model, some intermediate risk cases and the high risk group (36% of the cohort, median OS of 33 months) might be candidate to a rapid shift to second line therapies, investigational trials or even to allo-SCT (Table 3) [85].

**Table 3:** The Response to Ruxolitinib After 6 Months (RR6) model

Variable	Points
*RUX dose* < *20 mg BID at baseline, month 3, month 6*	1
≤ *30% spleen length reduction at month 3 and month 6*	1.5
*RBC units need at month 3 and/or month 6*	1
*RBC units need at baseline, month 3 and month 6*	1.5
Risk category, score (% of patients)	Overall survival, months
*Low, 0 (19%)*	Not reached
*Intermediate, 1–2 (45%)*	61
*High,* ≥ *2.5 (36%)*	33

RUX = ruxolitinib; BID = every 12 h; RBC = red blood cells

Some speculations could be done also on other JAKis. Progression-free survival looked significantly prolonged with fedratinib *vs* placebo in the JAKARTA trial [21, 94]. In the SYMPLIFY-1 study, there was an association between RBC-transfusion independence (TI) at 24 weeks and improved 3-year OS with momelotinib (MMB), suggesting RBC-TI as a potential surrogate for disease modification with this JAKi [95].

In phase 2 studies, some investigative drugs (added-on to RUX or alone) seem to be associated with benefit on OS, especially in case of “biological responses” (i.e., reduction of driver genes VAF, BMF grade or circulating CD34 + cells) [96–100]. Of course, definitive conclusions could be drawn only by randomized trials, by a long follow-up and ideally considering as primary endpoint either OS or potential surrogate markers [99]. Interestingly, changes in BMF grade at 6 months in SIMPLIFY-1 patients treated either with RUX or MMB did not correlate with OS, suggesting that the potential “disease-modifying” effect of a class of agents could be related to its specific mechanism of action [101].

## Conclusions

In the recent years, outcome of MF patients has improved, due to early diagnosis, use of JAKis and improved management of candidates to allo-SCT. Nonetheless, MF still remains a severe disease, that deserves an accurate prognostic definition.

The increased knowledge on the biological landscape of PMF has broadened the number of available survival models, that should be applied simultaneously for a more personalized definition of outcome, especially in younger patients.

The evidence that SMF is a specific entity has led to a more intensive monitoring of PV and ET cases for possible signs of progression. Moreover, we have now an ad hoc prognostic score, the MYSEC-PM, unanimously adopted by the NCCN and European guidelines. Integrated statistical methods will help to incorporate NGS results on SMF prognostication.

Fit MF patients with an estimated survival below five years are potential candidate to allo-SCT, but application of models such as the MTSS is required to predict post-transplant outcome and related complications.

Majority of MF patients are not suitable for allo-SCT and mostly receive JAKis. In RUX treated cases, the RR6 model is a useful tool to early identify subjects with reduced survival and that deserve a prompt treatment shift. There are some signs of survival benefit with RUX or innovative drugs in phase 2 studies, but we believe that more definitive evidence could be drawn only by designing trials with survival or its surrogate markers as primary endpoint.

## Key References

- Arber DA, Orazi A, Hasserjian R, Borowitz MJ, Calvo KR, Kvasnicka HS, et al. International Consensus Classification of Myeloid Neoplasms and Acute Leukemias: integrating morphologic, clinical, and genomic data. Blood. 2022;140(11):1200-228.
  - **WHO 2022 classification of myeloid malignancies.**
- Barosi G, Mesa RA, Thiele J, Cervantes F, Campbell PJ, Versovsek S, et al. Proposed criteria for the diagnosis of post-polycythemia vera and post-essential thrombocythemia myelofibrosis: a consensus statement from the International Working Group for Myelofibrosis Research and Treatment. Leukemia. 2008;22(2):437-38.
  - **International diagnostic criteria for secondary myelofibrosis.**
- Passamonti F, Mora B, Giorgino T, Guglielmelli P, Cazzola M, Maffioli M, et al. Driver mutations' effect in secondary myelofibrosis: an international multicenter study based on 781 patients. Leukemia. 2017;31(4):970-73.
  - **Distribution and correlations of driver mutations in the widest cohort of secondary myelofibrosis patients to date.**
- Luque Paz D, Kralovics R, Skoda RC. Genetic basis and molecular profiling in myeloproliferative neoplasms. Blood.2023;141(16):1909-921.
  - **Recent comprehensive review of genetic basis of myeloproliferative neoplasms.**
- Passamonti F, Cervantes F, Vannucchi AM, Morra E, Rumi E, Pereira A, et al. A dynamic prognostic model to predict survival in primary myelofibrosis: a study by the IWG-MRT (International Working Group for Myeloproliferative Neoplasms Research and Treatment). Blood. 2010;115(9):1703-8.
  - **Dynamic prognostic model for primary myelofibrosis.**
- Passamonti F, Giorgino T, Mora B, Guglielmelli P, Rumi E, Maffioli M, et al. A clinical-molecular prognostic model to predict survival in patients with post polycythemia vera and post essential thrombocythemia myelofibrosis. Leukemia. 2017;31(12):2726-31.
  - **Specific prognostic model for secondary myelofibrosis.**
- Guglielmelli P, Pacilli A, Rotunno G, Rumi E, Rosti V, Delaini F, et al. Presentation and outcome of patients with 2016 WHO diagnosis of prefibrotic and overt primary myelofibrosis. Blood. 2017;129(24):3227-36.
  - **Distinctions in presentation and survival between prefibrotic- and overt-primary myelofibrosis.**
- Tefferi A, Guglielmelli P, Larson DR, Finke C, Wassie EA, Pieri L, et al. Long-term survival and blast transformation in molecularly annotated essential thrombocythemia, polycythemia vera, and myelofibrosis. Blood. 2014;124(16):2507-13.
  - **Description of long term events in myeloproliferative neoplasms.**
- Masarova L, Bose P, Pemmaraju N, Daver NG, Sasaki K, Chifotides HT, et al. Improved survival of patients with myelofibrosis in the last decade: Single-center experience. Cancer. 2022;128(8):1658-65.
  - **Evolution of outcome in myelofibrosis over years.**
- Verstovsek S, Parasuraman S, Yu J, Shah A, Kumar S, Xi A, et al. Real-world survival of US patients with intermediate- to high-risk myelofibrosis: impact of ruxolitinib approval. Ann Hematol. 2022;101(1):131-37.
  - **Impact of current myelofibrosis treatments on outcome in myelofibrosis.**
- Verstovsek S, Gotlib J, Mesa RA, Vannucchi AM, Kiladjian JJ, Cervantes F, et al. Long-term survival in patients treated with ruxolitinib for myelofibrosis: COMFORT-I and -II pooled analyses. J Hematol Oncol. 2017;10(1):156.
  - **Post-hoc analysis on survival of COMFORT studies.**
- Pardanani A, Tefferi A, Masszi T, Mishchenko E, Drummond M, Jourdan E, et al. Updated results of the placebo-controlled, phase III JAKARTA trial of fedratinib in patients with intermediate-2 or high-risk myelofibrosis. Br J Haematol. 2021;195(2):244-48.
  - **Results of fedratinib in patients with myelofibrosis naïve to treatment.**
- Kröger N, Bacigalupo A, Barbui T, Ditschkowski M, Gagelmann N, Griesshammer M, et al. Indication and management of allogeneic haematopoietic stem-cell transplantation in myelofibrosis: updated recommendations by the EBMT/ELN International Working Group. Lancet Haematol. 2024;11(1):e62-74.
  - **Updated international guidelines on allogeneic haematopoietic stem-cell transplantation in myelofibrosis.**
- Guglielmelli P, Lasho TL, Rotunno G, Mudireddy M, Mannarelli C, Nicolosi M, et al. MIPSS70: Mutation-Enhanced International Prognostic Score System for Transplantation-Age Patients With Primary Myelofibrosis. J Clin Oncol. 2018;36(4):310-18.
  - **Molecularly-annotated prognostic score for primary myelofibrosis patients potentially candidates to allogeneic haematopoietic stem-cell transplantation.**
- Tefferi A, Guglielmelli P, Lasho TL, Gangat N, Ketterling RP, Pardanani A, et al. MIPSS70+ Version 2.0: Mutation and Karyotype-Enhanced International Prognostic Scoring System for Primary Myelofibrosis. J Clin Oncol. 2018;36(17):1769-70.
  - **Integration of molecular and cytogenetic data in a prognostic score for primary myelofibrosis patients potentially candidates to allogeneic haematopoietic stem-cell transplantation.**
- Tefferi A, Guglielmelli P, Nicolosi M, Mannelli F, Mudireddy M, Bartalucci N, et al. GIPSS: genetically inspired prognostic scoring system for primary myelofibrosis. Leukemia. 2018;32(7):1631-42.
  - **Prognostic score only based on biology for primary myelofibrosis.**
- Network NCC. Myeloproliferative neoplasms. 2024 [Available from https://www.nccn.org/professionals/physician_gls/pdf/mpn.pdf]
  - **Current NCCS guidelines for myelofibrosis.**
- Luque Paz D, Riou J, Verger E, Cassinat B, Chauveau A, Ianotto JC, et al. Genomic analysis of primary and secondary myelofibrosis redefines the prognostic impact of ASXL1 mutations: a FIM study. Blood Adv. 2021;5(5):1442-51.
  - **Analysis of a large cohort of myelofibrosis patients annotated for NGS.**
- Guglielmelli P, Coltro G, Mannelli F, Rotunno G, Loscocco GG, Mannarelli C, et al. ASXL1 mutations are prognostically significant in primary myelofibrosis, but not myelofibrosis following essential thrombocythemia or polycythemia vera. Blood Adv. 2022;6(9):2927-31.
  - **Discussion of the prognostic role of ASXL1 in primary and secondary myelofibrosis.**
- Harrison CN, Nangalia J, Boucher R, Jackson A, Yap C, O’Sullivan J, et al. Ruxolitinib Versus Best Available Therapy for Polycythemia Vera Intolerant or Resistant to Hydroxycarbamide in a Randomized Trial. J Clin Oncol. 2023;41(19):3534-44.
  - **Prospective randomized trial of ruxolitinib compared to best available therapy in patients with polycythemia vera after hydroxycarbamide.**
- Mora B, Giorgino T, Guglielmelli P, Rumi E, Maffioli M, Rambaldi A, et al. Value of cytogenetic abnormalities in post-polycythemia vera and post-essential thrombocythemia myelofibrosis: a study of the MYSEC project. Haematologica. 2018;103(9):e392-94.
  - **Description of cytogenetic alterations in a large cohort of secondary myelofibrosis.**
- Gupta V, Malone AK, Hari PN, Woo Ahn K, Hu ZH, Gale RP, et al. Reduced-intensity hematopoietic cell transplantation for patients with primary myelofibrosis: a cohort analysis from the center for international blood and marrow transplant research. Biol Blood Marrow Transplant. 2014;20(1):89-97.
  - **Outcome of reduced-intensity hematopoietic cell transplantation in primary myelofibrosis.**
- Passamonti F. Stem cell transplant in MF: it's time to personalize. Blood. 2019;133(20):2118-20.
  - **Discussion on the indications and guidance on selection of myelofibrosis patients for stem cell transplant.**
- Gagelmann N, Ditschkowski M, Bogdanov R, Bredin S, Robin M, Cassinat B, et al. Comprehensive clinical-molecular transplant scoring system for myelofibrosis undergoing stem cell transplantation. Blood. 2019;133(20):2233-42.
  - **Integrated prognostic score for selection of myelofibrosis patients for stem cell transplant.**
- Tamari R, McLornan DP, Ahn KW, Estrada-Merly N, Hernández-Boluda JC, Giralt S, et al. A simple prognostic system in patients with myelofibrosis undergoing allogeneic stem cell transplantation: a CIBMTR/EBMT analysis. Blood Adv. 2023;7(15):3993-4002.
  - **Semplified prognostic score for selection of myelofibrosis patients for stem cell transplant.**
- Guglielmelli P, Ghirardi A, Carobbio A, Masciulli A, Maccari C, Mora B, et al. Impact of ruxolitinib on survival of patients with myelofibrosis in the real world: update of the ERNEST Study. Blood Adv. 2022;6(2):373-75.
  - **Real world data on survival of myelofibrosis patients treated with ruxolitinib or hydroxycarbamide.**
- Maffioli M, Mora B, Ball S, Iurlo A, Elli EM, Finazzi MC, et al. A prognostic model to predict survival after 6 months of ruxolitinib in patients with myelofibrosis. Blood Adv. 2022;6(6):1855-64.
  - **Prognostic score for evaluating survival after 6 months of ruxolitinib.**
- Kuykendall AT, Shah S, Talati C, Al Ali N, Sweet K, Padron E, et al. Between a rux and a hard place: evaluating salvage treatment and outcomes in myelofibrosis after ruxolitinib discontinuation. Ann Hematol. 2018;97(3):435-41.
  - **Evaluation of incidence and reasons of ruxolitinib discontinuation and its outcome.**
- Pemmaraju N, Garcia JS, Potluri J, Harb JG, Sun Y, Jung P, et al. Addition of navitoclax to ongoing ruxolitinib treatment in patients with myelofibrosis (REFINE): a post-hoc analysis of molecular biomarkers in a phase 2 study. Lancet Haematol. 2022;9(6):e434–44.
  - **Post-hoc analysis of impact of changes in molecular biomarkers on outcome of patients treated with ruxolitinib and navitoclax.**
- Mascarenhas J, Komrokji RS, Palandri F, Martino B, Niederwueser D, Reiter A, et al. Randomized, single-blind, multicenter phase II study of two doses of Imetelstat in relapsed or refractory myelofibrosis. J Clin Oncol. 2021;39(26):2881–892.
  - **Results of imetelstat in relapsed/refractory myelofibrosis patients.**
- Pemmaraju N, Verstovsek S, Mesa R, Gupta V, Garcia JS, Scandura JM, et al. Defining disease modification in myelofibrosis in the era of targeted therapy. Cancer. 2022;128(13):2420-32.
  - **Definition of disease modification in myelofibrosis in the contemporary era.**

## Data Availability

No datasets were generated or analysed during the current study.
